# Targeted Cerebral Oxygenation Using Dedicated Treatment Versus Usual Care in Extremely Preterm Infants: Protocol for a Multicentre International Phase II Randomised Controlled Trial

**DOI:** 10.1111/jpc.70066

**Published:** 2025-04-21

**Authors:** Pranav R. Jani, Traci‐Anne Goyen, Kiran Kumar Balegar Virupakshappa, Rajesh Maheshwari, Dharmesh Shah, Maria Saito‐Benz, Tim Schindler, James Moore, James Elhindi, Himanshu Popat, NIRTURE Trial Group

**Affiliations:** ^1^ Department of Neonatology Westmead Hospital Westmead Australia; ^2^ Faculty of Medicine and Health The University of Sydney Sydney Australia; ^3^ Department of Neonatology Nepean Hospital, Nepean Blue Mountains Local Health District Kingswood Australia; ^4^ Sydney Medical School Nepean The University of Sydney Sydney New South Wales Australia; ^5^ Neonatal Intensive Care Unit Wellington Regional Hospital Wellington New Zealand; ^6^ Department of Paediatrics and Child Health University of Otago Wellington New Zealand; ^7^ Department of Newborn Care The Royal Hospital for Women Sydney Australia; ^8^ School of Clinical Medicine University of New South Wales Sydney Australia; ^9^ Connecticut Children's, Division of Neonatal‐Perinatal Medicine Connecticut Children's Medical Center Hartford Connecticut USA; ^10^ UCONN School of Medicine Farmington Farmington Connecticut USA; ^11^ Research and Education Network Westmead Hospital Westmead Australia; ^12^ Grace Centre for Newborn Intensive Care The Childre's Hospital for Westmead Westmead Australia; ^13^ NHMRC Clinical Trial Centre University of Sydney Camperdown Australia

**Keywords:** cerebral oxygenation, extremely preterm infants, near infra‐red spectroscopy, randomised controlled trial

## Abstract

**Background:**

Near infrared spectroscopy (NIRS) allows continuous monitoring of cerebral oxygenation and therefore has the potential to be neuroprotective. Recurrent episodes of cerebral hypo‐and/or hyperoxia may result in brain injury. The Safe‐BoosC‐II study reported stable cerebral oxygenation in extremely preterm infants by combining a dedicated treatment guideline with NIRS monitoring using several devices and adult sensors. The ability to maintain stable cerebral oxygenation with a dedicated treatment algorithm using one type of NIRS device with neonatal sensors has not been previously investigated.

**Methods:**

In this multicentre, 2‐arm, parallel, single‐blinded, phase II RCT, stratified by gestation and hospital site, 100 participants born < 29 weeks' gestation (inborn and outborns, singleton and twins) will be randomised to targeted cerebral oxygenation using dedicated treatment or usual care with blinded cerebral NIRS monitoring for the first 5 days of life. We will exclude infants > 6 h of age, those with congenital anomaly requiring major surgery or a genetic disorder, and triples or higher multiple births. The primary outcome is the burden of cerebral hypoxia and hyperoxia for the first 5 days after birth expressed as percent hours.

**Discussion:**

The findings of this trial will provide essential information on (i) validating results from the Safe‐BoosC‐II study, considering the differences in the study methodology between the two trials (ii) strengthening support for routine use of cerebral NIRS monitoring in this population and (iii) informing the design of future RCTs on the effects of targeted cerebral oxygenation on neurodevelopment in early childhood as the primary outcome.

**Trial Registration:**

Australian New Zealand Clinical Trials Registry registration number ACTRN12621000778886

**Abbreviations**
CrSO_2_
: cerebral oxygenation
DMSC: Data Safety and Management Committee
FiO_2_
: fraction of inspired oxygen
GEE: generalised estimating equation
NICU: neonatal intensive care unit
NIRS: near infrared spectroscopy
NIRTURE: near infra‐red spectroscopy targeted use to reduce adverse outcomes in extremely preterm infants
PCO_2_
: partial pressure of carbon dioxide in the blood
PMA: postmenstrual age
RCT: randomised controlled trial
SpO_2_
: peripheral oxygen saturation
SVC: superior vena cava

## 1 Background

Premature infants compared to term‐born infants are at a greater risk of death, cerebral palsy, intellectual impairment, learning disorders, vision, and hearing loss [1, 2, 3]. There are significant financial and economic impacts when caring for a child with cerebral palsy [4].

One proposed mechanism for brain injury is exposure to periods of low oxygen (hypoxia) and high oxygen (hyperoxia). Large randomised controlled trials comparing lower (85%–89%) versus higher (91%–95%) peripheral saturation target ranges in extremely preterm infants found no differences in neurodevelopment, including cerebral palsy [5, 6, 7, 8, 9, 10, 11]. While these studies targeted whole body saturation, a physiological approach would be to target cerebral oxygenation.

**FIGURE 1:**
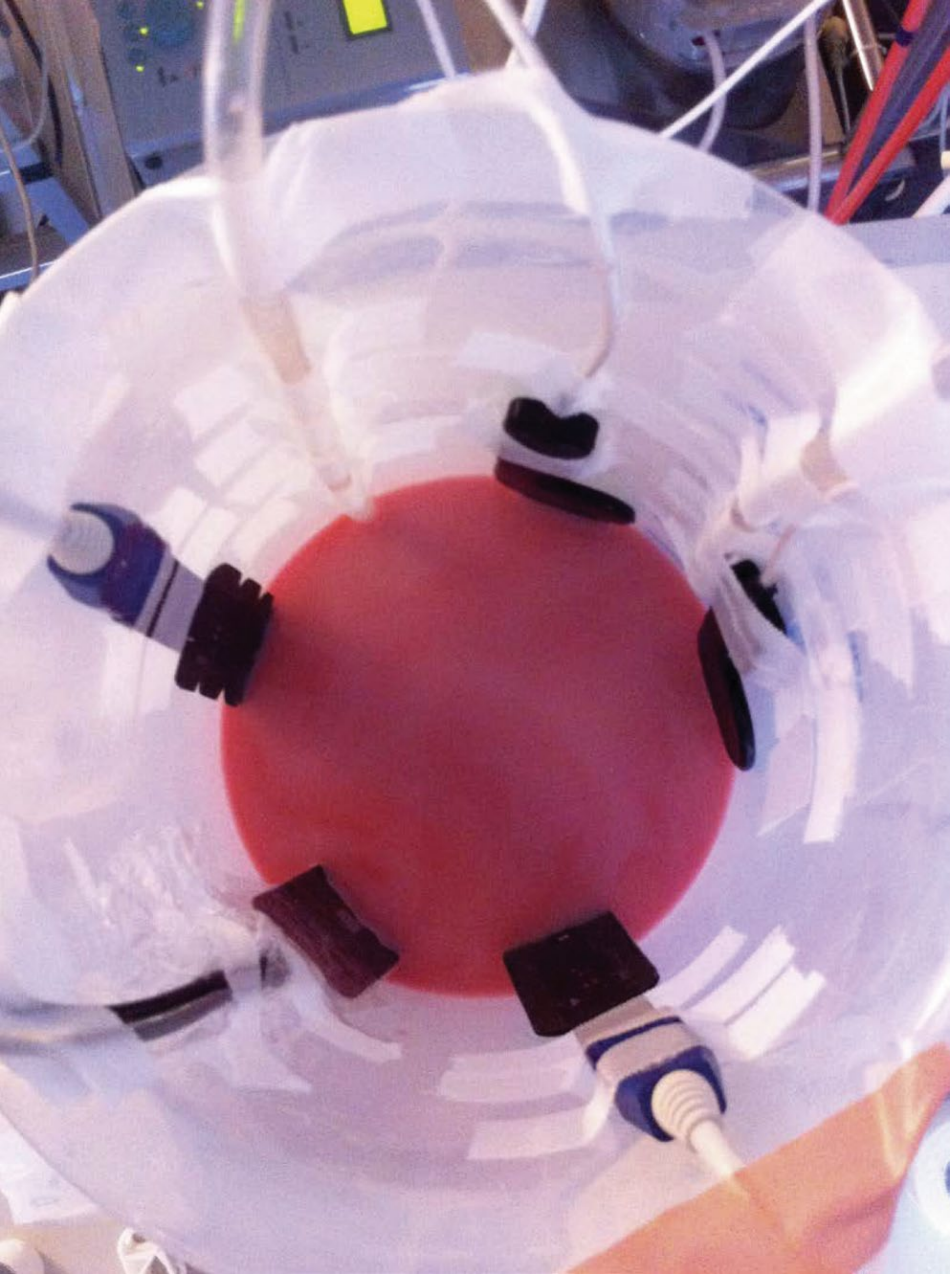
The blood‐lipid phantom for calibrating oximeter sensors.

Cerebral near infra‐red spectroscopy (NIRS) monitoring allows assessment of cerebral oxygenation [12]. The SafeBoosC‐II study found cerebral NIRS monitoring in combination with a dedicated clinical treatment guideline compared with blinded cerebral NIRS monitoring and treatment as usual, reduced the burden of hypoxia and hyperoxia from 81% to 36% hours during the first 3 days of life (*p* < 0.001) [13]. However, they used several types of NIRS devices with adult sensors that were calibrated against the INVOS adult sensor by using a blood‐lipid phantom to determine the device specific intervention thresholds of 55% and 85% (Figure 1) [14]. Cerebral oxygenation levels differ for different types of NIRS devices and the type of sensor (adult vs. newborn) [15]. Additionally, the devices were modified to sound an alarm when the cumulative burden of hypo and/or hyperoxia reached a burden of 0.2% hours (12% min) had accumulated during the past 10 min [13].

The current trial (Near Infra‐Red Spectroscopy Targeted Use to Reduce adverse outcomes in Extremely preterm infants: NIRTURE) differs from the SafeBoosC‐II study by using one type of NIRS device and a neonatal sensor, intervention duration of 5 days of life to investigate the burden of cerebral hypoxia and hyperoxia as the primary outcome. Before we undertake a large multisite international RCT investigating the impact of using a combination of cerebral NIRS monitoring and dedicated clinical treatment guideline on neurodevelopment in early childhood as the primary outcome [16], it is important to conduct a phase‐II RCT incorporating important differences to the SafeBoosC–II study.

## 2 Methods/Design

### 2.1 Study Setting

This trial will include five tertiary NICUs across Australia, New Zealand, and the US with investigator experience in cerebral NIRS monitoring. Ethical approvals have been obtained in all three countries before commencement (For Australian sites: The Sydney Children's Hospitals Network Human Research Ethics Committee: 2020/ETH02903, For New Zealand site: The Health and Disability Ethics Committee approved the trial in New Zealand: 21/NTB/157 and for US site: Institutional Review Board of the Connecticut Children's Medical Center: IRB 21‐071). The trial's conduct will align with the International Council for Harmonisation guidelines for Good Clinical Practice. Clinical staff at all sites will undergo training and web‐based certification before commencing participant recruitment.

### 2.2 Objective

The primary objective of the NIRTURE trial is to ascertain whether the combination of a dedicated treatment along with cerebral oxygenation monitoring compared to usual treatment with blinded NIRS monitoring, reduces the burden of cerebral hypoxia (CrSO_2_ < 65%) and hyperoxia (CrSO_2_ > 90%) in the first 5 days after birth in premature infants born < 29 weeks gestation. We selected this study population for the following reasons: across neonatal intensive care units (NICUs) in Australia and New Zealand, they have a combined adverse outcome incidence of ~33% [17], a higher risk of cerebral palsy (12% vs. 1% in term born) [18] and there are published reference values of cerebral oxygenation ranges during the transitional phase after birth [19]. We hypothesise that the burden of hypoxia and hyperoxia may be reduced by a combination of dedicated treatment algorithm with cerebral oxygenation monitoring when using a single NIRS device and neonatal sensor. This trial will be a single‐blinded RCT with two parallel groups with 1:1 allocation stratified for site and gestational age (< 26 weeks and ≥ 26^+0^–28^+6^ weeks' gestation).

### 2.3 Eligibility Criteria

This trial will include inborn or outborn infants (singleton or twin births) born < 29 weeks' gestation and < 6 h of age. We will exclude infants admitted > 6 h of age, those with an antenatal or postnatal diagnosis of congenital anomaly requiring major surgery or a known genetic disorder associated with neurological impairment, and multiple births beyond twins.

### 2.4 Interventions

**FIGURE 2:**
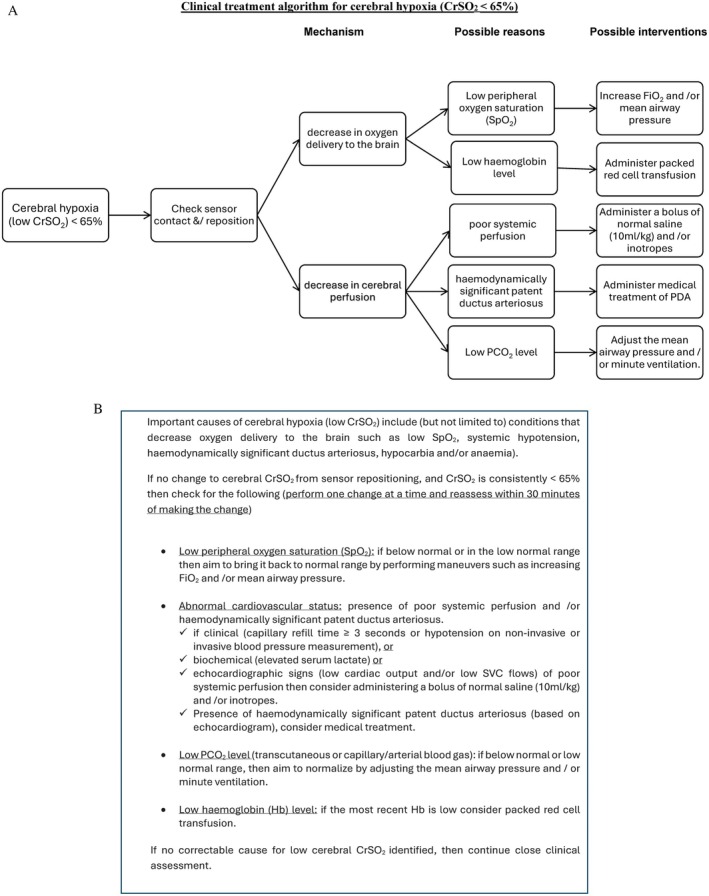
(A) Clinical treatment algorithm for cerebral hypoxia. (B) Interventions for cerebral hypoxia. CrSO_2_: Cerebral oxygenation, Hb: Haemoglobin, SpO_2_: Peripheral oxygen saturation, SVC: Superior vena cava.

**FIGURE 3:**
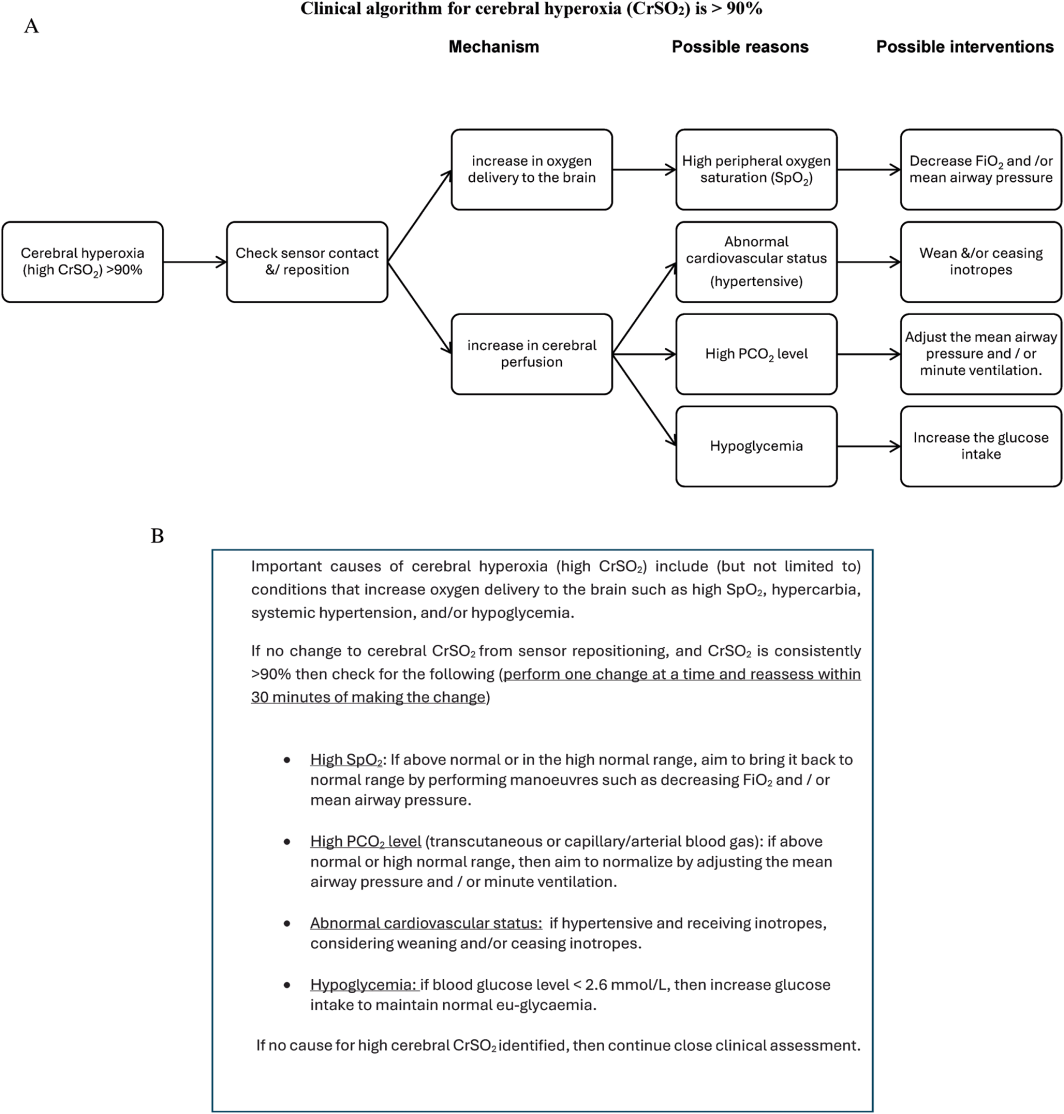
(A) Clinical algorithm for cerebral hyperoxia. (B) Interventions for cerebral hyperoxia. CrSO_2_: Cerebral oxygenation, PCO_2_: Partial pressure of carbon dioxide in blood, SpO_2_: Peripheral oxygen saturation.

After enrolment, infants will be randomised to either the control group or the intervention group. Monitoring of cerebral oxygenation will be commenced by using the SenSmart Model X‐100 Universal Oximetry System (from NONIN Medical Inc., MN, USA) and placing a neonatal NIRS sensor on the fronto‐parietal region of the infant's forehead, and monitoring will be continued for 5 days (120 h of monitoring). For the Intervention group, cerebral oxygenation reading will be visible, and the infant will be treated according to a dedicated clinical treatment algorithm when the cerebral oxygenation is outside the range of 65%–90% (Figures 2 and 3).

#### 2.4.1 Intervention Thresholds

A pragmatic consensus‐based approach was used for selecting 65%–90% as the reference range for cerebral oxygenation. The lower threshold of 65% was chosen based on the lower threshold (55% using an adult sensor) used by the SafeBoosC‐II study and higher absolute oxygenation values with neonatal sensors [15]. An upper threshold value of 90% instead of 85% (as selected by the SafeBoosC‐II study) was chosen from our local, unpublished data by monitoring preterm infants born < 28 weeks gestation in the first 3 days of life with NONIN neonatal sensors (cerebral oxygenation of 87% was the 75th centile). The sensor site will be inspected every 4 h to ensure correct sensor placement and skin integrity. Bedside clinicians will receive training on the application of NIRS monitoring, troubleshooting advice for sensor disconnection and for technical issues.

A clinical treatment algorithm for cerebral hypoxia is triggered when cerebral oxygenation, CrSO_2_, is < 65% (Figure 2). When Cerebral oxygenation (CrSO_2_) is < 65%, potential causes include inaccurate sensor placement, low arterial oxygen saturation [20, 21, 22, 23], reduced cerebral blood flow [23, 24, 25, 26, 27, 28, 29, 30, 31, 32, 33, 34, 35, 36, 37, 38] or reduced haemoglobin concentration in circulating blood [39, 40, 41, 42, 43]. A clinical treatment algorithm for cerebral hyperoxia is triggered when cerebral oxygenation, CrSO_2_, is > 90% (Figure 3). When cerebral oxygenation (CrSO_2_) is > 90%, potential causes include inaccurate sensor placement, high arterial oxygen saturation [7, 8, 9, 10, 20, 21, 44, 45, 46, 47], increased cerebral blood flow [24, 25, 35, 38], or hypoglycaemia [48, 49].

For the control group, cerebral oxygenation reading will not be visible to the bedside staff and the infant will be treated according to standard clinical practice. We will perform blinded NIRS monitoring by covering the monitor screen with an opaque cover, and the information will not be available to clinicians to act upon.

### 2.5 Modifications

The intervention will be discontinued if requested by parents or clinicians.

### 2.6 Adherence

For the intervention group: A bedside “Response to Alarm” form will be made available for staff to prospectively record actions performed for low and high cerebral oxygenation alarms to ensure compliance to the clinical algorithm.

For the control group: A bedside “Response to alarm” form will be made available for staff to record prospectively actions performed for sensor displacement alarms. There are no restrictions on concomitant medications or care practices.

### 2.7 Outcomes

Primary outcome: The burden of cerebral hypoxia and hyperoxia during the first 5 days after birth expressed as percent hours. For example, a 1‐h event with mean cerebral oxygenation of 55% would be 10% hours (10% below the lower threshold of 65% multiplied by 1 h).

### 2.8 Secondary Outcomes

- Mortality before hospital discharge
- Brain injury based on imaging (all grades of intraventricular haemorrhage or cerebellar haemorrhage [50] or periventricular leukomalacia) before hospital discharge
- Neonatal morbidities (individual reporting of the incidence of chronic lung disease‐need for respiratory support or supplemental oxygen at 36 weeks' postmenstrual age, necrotising enterocolitis using Bell's classification [51] and retinopathy of prematurity) prior to hospital discharge
- Physiological functions (observation of pulse oximetry–heart rate and peripheral oxygen saturation—SpO_2_) at 36–37 weeks' postmenstrual age (PMA)
- Sleep architecture (amount and distribution of various stages of sleep, based on sleep study) at 36–37 weeks' PMA (at two sites)
- General movements assessment during the writhing and the fidgety stages
- Age‐appropriate standardised neurodevelopmental outcomes until 5 years of age (Cognition, motor and language domain scores on Bayley Scales of Infant and Toddler development 4th Edition or the Wechsler Preschool and Primary Scale of Intelligence)
- Measurement of physiological functions (heart rate, SpO_2_, blood pressure) in the first 5 days after birth

Safety outcome: Skin injury (pressure or thermal) from NIRS sensor in the first 5 days after birth and adverse event reporting.

**FIGURE 4:**
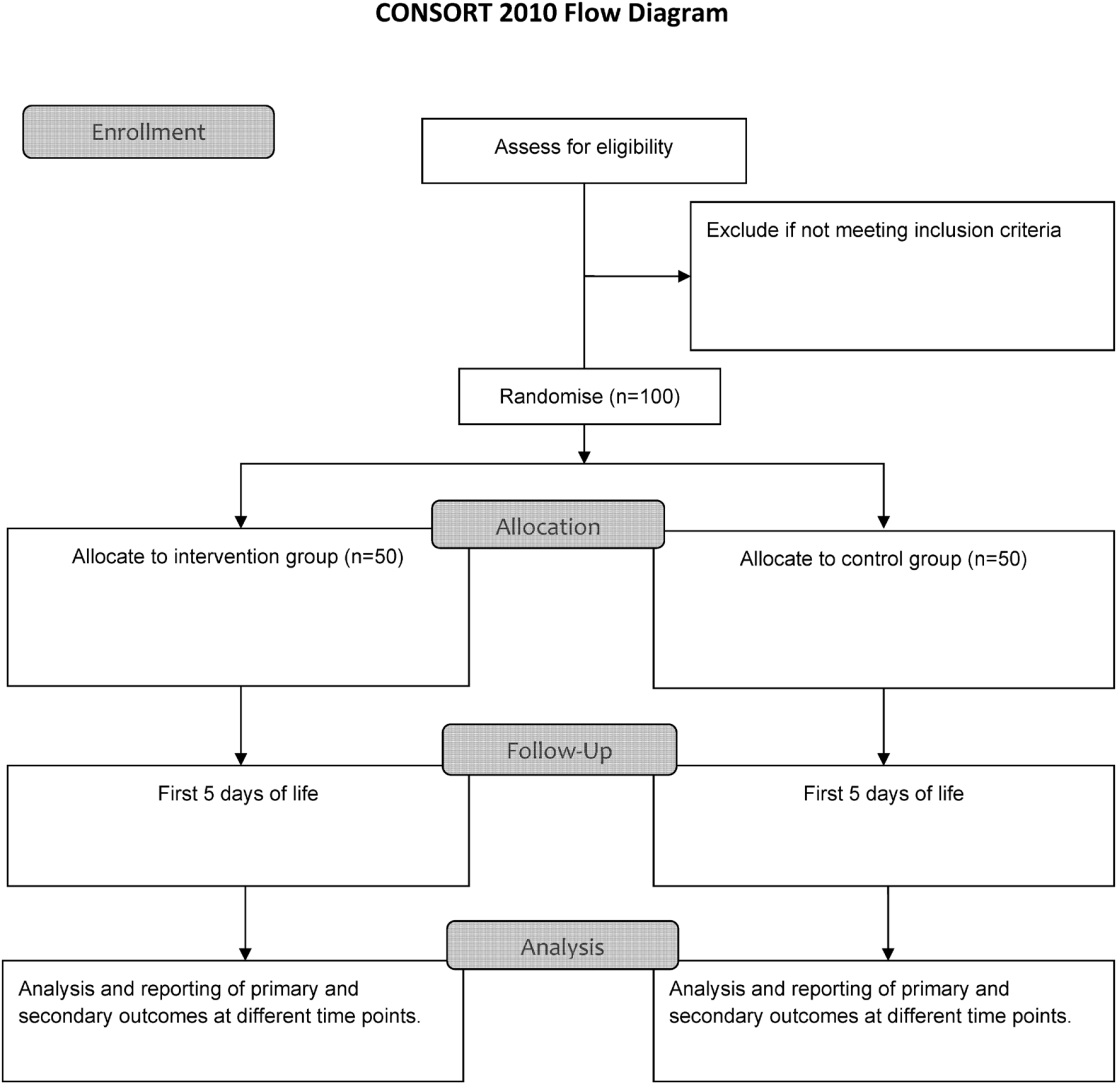
CONSORT flow diagram.

Participant timeline for the study is shown in Table 1 and Figure 4.

**TABLE 1:** Study timeline for enrolled participants.

Timepoint	Enrolment	Allocation of intervention	Study period								
			T1	T2	T3	T4	T5	T6	T7	T8	T9
**Enrolment**											
Eligibility screening	**X**										
Informed consent	**X**										
Allocation		**X**									
**Intervention**											
Intervention group			**X**								
Control group			**X**								
**Assessments**											
Skin integrity tool			**X**								
Response to alarm record			**X**								
Cranial ultrasounds				**X**	**X**						
Overnight pulse oximetry						**X**					
Day sleep study (once)						**X**					
MRI brain (if performed)							**X**				
Motor performance						**X**		**X**			
Neurodevelopment follow‐up								**X**	**X**	**X**	**X**

Abbreviations: T1 = post‐allocation to 5 days from birth; T2 = first week from birth; T3 = 32 weeks PMA or 6 weeks after birth; T4 = 36–37 weeks PMA; T5 = term PMA (37–40 weeks); T6 = Follow‐up assessment at 3–4 months corrected age; T7 = 12 months corrected age; T8 = 18–24 months corrected age; T9 = 5 years of age.

### 2.9 Sample Size

Based on the results of the Safe‐BoosC‐II study [13], a 50% reduction in mean burden of hypoxia and hyperoxia in active treatment relative to control would be considered worthwhile. This corresponds to a reduction of 0.3 in the mean of log‐transformed burden of cerebral hypoxia and/or hyperoxia. In the Safe‐BoosC‐II study, a 58% reduction was achieved. Assuming a standard deviation of 0.5 in log‐transformed burden [52], this would require 45 babies per group to achieve 80% power at 5% two‐sided alpha. To account for the clustering effect of twins being allocated to the same treatment, we assume that 30% of babies are twins‐based on regional incidence of twin births (giving an average cluster size of 1.3), and a within‐twin correlation of 0.1 in the outcome. This gives a design effect of 1.03 by which the sample size is adjusted, leading to a sample size of 47 per group, 94 babies in total. We aim to recruit 100 babies to account for some loss to follow‐up or missing data. For consented participants, if the NIRS monitoring was discontinued earlier than 5 days after birth, data collected until discontinuation of monitoring will be used for analysis of the primary outcome.

### 2.10 Recruitment

Recruitment of participants will occur after obtaining an informed written consent (USA and New Zealand) or using a deferred consent (for Australian sites only). An informed written consent will be obtained from the parent in the antenatal period or postnatally within 6 h of birth. Where antenatal consent could not be obtained, a deferred consent has been approved for enrolment of the baby (for Australian sites only). Written consent will be obtained prior to using data, and if consent has not been obtained then the data will not be used.

Where a participant was randomised but consent could not be obtained in the first 5 days after birth (due to death or development of a life‐threatening complication in the infant), no attempt will be made to contact the parents for a consent and all data collected so far will be removed from the study. Should grieving parents accept continuation of intensive care treatment, then contact will be made with the parents when they are in a state of being approached for consent.

Additionally, where parent/s are isolated from having COVID‐19 or other viral infections, contact will be made with the parents when they are in a state of being approached for consent, which will usually be beyond 5 days of age.

### 2.11 Assignment of Interventions

#### 2.11.1 Sequence Generation

Central randomisation will be performed by the site investigator or the medical team member using REDCap electronic data capture tools hosted at the University of Sydney. Infants assessed as eligible will be randomised to the control group or intervention group by using variable block randomisation stratified for gestational age (< 26 weeks' and ≥ 26 weeks') and study site. Randomisation codes for the study will be generated by a biostatistician independent of the study. Permuted blocks will maintain group balance; infants will be allocated to two groups in a 1:1 ratio. Twins will be randomised to the same group, based on the negligible intra‐class coefficient burden of hypoxia within pairs of twins in the SafeBoosC‐II study [13] and the survey of parents of multiple births [53].

To ensure concealment, the block sizes will not be disclosed. Additionally, allocation concealment will be ensured, as REDCap will not reveal the randomisation until the infant has been recruited into the trial. REDCap will maintain a list of participants enrolled. Each site investigator will access only their site records. The chief investigators will have access to all sites to oversee enrolment. Participants will be given unique codes to protect their identity on all documentation utilised for recording and analysis purposes. Separate data records will be maintained for re‐identifiable and de‐identified data.

#### 2.11.2 Blinding (Masking)

During the intervention period, the assignment of infants to the control arm or intervention arm cannot be blinded to the clinical staff. For infants in the control arm, blinding will be performed by covering the NIRS monitor screen with an opaque cover. Also, alarms for the range of cerebral oxygenation (65%–90%) will be turned off and alarms for displacement/dislodgement of the sensors will remain active. The statistician and neurodevelopment outcome assessors will be blinded to the intervention.

### 2.12 Data Analysis and Management

Participant demographic and clinical characteristics and study outcomes will be presented using standard descriptive statistics: frequencies and percentages for categorical variables, mean, standard deviation and range or median, quartiles and range for continuous variables and the Kaplan–Meier or similar method for time‐to‐event variables. All efficacy analyses will be performed according to randomised treatment. We will use a suitable transformation to ensure normality for the primary outcome, the burden of cerebral hypoxia and hyperoxia, and use generalised estimating equation (GEE) or a similar method for comparison between groups. Sub‐group analysis for stratification variables will be performed. For reporting the burden of cerebral hypoxia and hyperoxia, we will include deviations in cerebral oxygenation of more than a minute. This pragmatic approach will ensure we achieve a balance between responding to an alarm from poor sensor contact with the participant and identifying true real‐time changes in cerebral oxygenation. To allow comparison of the primary outcomes with other studies, we will also report the burden of cerebral hypoxia and hyperoxia of more than 10 min. Other study outcomes will be compared using similar methods, using GEE methods with normal or binary outcome distribution and correlation between outcomes in twins. Adjusted models will explore predictors of outcomes.

Withdrawn participants will be replaced in the study with new randomisation (modified intention‐to‐treat analysis). To facilitate and ensure long‐term follow‐up, existing measures which are standard practices include providing appointment letters by post, reminding and confirming with families by phone and/or text message when follow‐up appointment is close to the date.

This trial will be conducted in accordance with applicable Privacy Acts and Regulations in all three countries. Data will be stored for a minimum of 15 years or until the youngest participant turns 25 (whichever is the longest).

### 2.13 Data Monitoring

An independent Data Safety and Management Committee (DSMC) comprising two clinicians and a biostatistician will be established to monitor the progress of all aspects of the study to ensure that the study meets the highest standards of ethics and patient safety. A charter will be written and agreed upon by the Trial Management Committee and DSMC for stopping the study prematurely, monitoring the trial more frequently or modifying the trial design (Supporting Information). The DSMC will meet every 6 months until completion of recruitment to identify safety issues.

### 2.14 Consumer Involvement

The NIRTURE study has involved consumers with a lived experience of preterm birth for reviewing and advising the study methodology, the consent process including advocating for deferred consent and the participant consent forms. They will also be involved with disseminating the results of the trial.

## 3 Discussion

The design of this trial will extend the validation of the findings of the landmark SafeBoosC‐II trial [13] by incorporating NIRS monitoring using a single device type with neonatal sensors that are appropriate for monitoring neonates. Our method for selecting cerebral hypoxia and hyperoxia thresholds for the intervention is pragmatic in nature, reflecting real‐world settings differing from those of the SafeBoosC‐III study, which used a blood‐lipid phantom to determine hypoxia thresholds for neonatal sensors [54]. The upper threshold of 90% was chosen to strike a pragmatic balance between excessive alarm triggering and identifying genuinely raised cerebral oxygenation values. Pre‐trial educational sessions are planned for training clinical staff on cerebral NIRS monitoring–reflecting an intervention under real‐world conditions. We chose 6 h of postnatal age cut‐off for participants' inclusion in the trial based on feedback from clinicians and consumers, and from work published by our group from another trial to allow explicit postnatal consenting at participating sites where deferred consent is not applicable [55]. The inclusion of major neonatal morbidities, rigorous data on General Movements, and neurodevelopment assessments will strengthen the design of future RCTs evaluating the impact of combining NIRS monitoring with a dedicated treatment algorithm as the intervention on neurodevelopment in childhood as a primary outcome. The study design, early consumer engagement in co‐designing the study, and the findings will strengthen the support for identifying cerebral hypoxia and hyperoxia using cerebral NIRS monitoring in the first 5 days after birth in this vulnerable population. Other trials such as the BOX trial (NCT05171881, a prospective, multicenter intervention trial) will provide opportunities for future individual patient data prospective meta‐analyses to demonstrate better healthcare outcomes. A limitation of the current trial is the exclusion of triplets and higher multiple births due to the number of NIRS devices available to deliver the intervention. We plan to disseminate the results via presentations, peer‐reviewed journal publications, and social media.

## Author Contributions

P.R.J. is the Principal Investigator. P.R.J. conceived the study, drafted the study protocol, obtained funding, coordinated ethics and legal approvals, managed data collection and storage process. H.P. conceived the study, assisted with providing intellectual contributions to the study protocol and study design, funding application, data collection and storage process. T.A.G. assisted with providing intellectual contributions to the study protocol and study design, funding application, data collection and storage process. R.M., K.K.B.V., M.S.B., T.S., D.S. and J.M. coordinated ethics and legal approvals, provided intellectual contribution to the study protocol, enrolled participants, managed data collection and storage. J.E. will perform data analysis and interpretation of the results. All authors reviewed and approved the final manuscript.

## Conflicts of Interest

The authors declare no conflicts of interest.

## Supporting information


**Data S1** Supporting Information.
